# Notes and News

**Published:** 1895-09-21

**Authors:** 


### Notes and News.

(Collected and Communicated.)

Professor Von Anrep has been appointed Director of the Women's Medical Institute, to be opened in St. Petersburg in 1897.

In an article on tuberculosis Dr. Ernest Martin, doctor to the French Legation in China, states that the Chinese are less subject to tuberculosis than other nations.

The Caxton Convalescent Home, for the benefit of all branches of the printing trades—situate at Limpsfield, Surrey, will be opened on Saturday, the 28th inst., by Mr. J. Passmore Edwards, the president, by whose munificence it has been erected.

The directors of the Aberdeen Royal Infirmary have resolved on the erection of a new convalescent home at Hillhead, Pitfodels. The home is to accommodate 25 patients. The funds in hand are £5,000 from the trustees of the late Mr. J. G. Chalmers, and the proceeds of site and buildings of the old home.

A good deal of indignation has been aroused by the creation of a medical school at Rennes. It is argued that another school so near to Nantes is unnecessary and derogatory to the prestige of French medical schools, seeing that the students at Rennes are very limited in number.

France is awakening to the fact that its towns are not sufficiently supplied with public baths. The important part which hydropathy plays in curative science nowadays is well recognised, and before long it may be expected that French municipalities will consider themselves incomplete without an establishment of public baths. In America Mr. Goodwin Brown, of Albany, secured the passing of an Act in New York State which rendered the establishment of free public baths compulsory in every large city of the State.

Amongst the troops invalided after the New Forest manœuvres, abrasions of the feet are the most common ailment.

We have good news for hospital secretaries. During the last week we have been consulted by two different sets of people who wished to know how to give away £14,000 to hospitals and kindred charities. We have always felt that the Hospital Sunday Fund afforded the best distributing centre for all who have no personal knowledge of the relative needs and merits of the various hospitals. But in compliance with the urgent request of those who consulted us, we have tried to indicate certain institutions which have special claims upon the benevolent. It is gratifying to be able to give this practical evidence of the desire to help the hospitals which exists at the present time in the minds of many people who have made large sums of money recently in the City and elsewhere. Hospital secretaries will, we hope, find once more that the last three months of the year are the most profitable in yielding funds for the maintenance of the institutions for the management of which they are responsible.

On Tuesday, July 3rd, the annual meeting of the Hospital Saturday Fund of Sydney was held. The Mayor of Sydney, Alderman S. E. Lees, M.L.A. (president of the fund), occupied the chair. The total income for the financial year ending May 4th last was £3,547 12s. 6d., compared with £1,948 4s. 11d. for the previous financial year. A division of the income for two years into indoor and outdoor collections shows the following: Indoor—1893-94, £523 2s. 3½d.; 1894-95, £1,259 4s. 9d. Outdoor—1893-94, £1,426 2s. 7½d.; 1894-95, £2,288 7s. 9d. The Lieutenant-Governor, Sir Frederick Darley, then distributed the cheques, which had been apportioned in conformity with the decision of the Distribution Committee as follows: Sydney Hospital, £1,000; Prince Alfred Hospital, £660; St. Vincent's Hospital, £450; Glebe Children's, £325; Carrington, £175; Parramatta Hospital, £140; North Shore Hospital, £140; Lewisham Hospital, £125; Thirlmere Hospital, £100; St. Joseph's Sanatorium, £60; Western Suburbs Hospital, £60; St. George's Hospital, £25.

The new wing of the Wigan Infirmary was formally opened with much ceremony and rejoicing on Wednesday, in last week, by Lady Maud Wilbraham, daughter of the Earl of Lathom. The day was fortunately fine, and the infirmary and grounds were thronged with some 1,500 guests during the day. The new building, erected through the generosity of the late Miss Amelia Gidlow, partly before her death in memory of her sister, and from legacies left by her, has been named appropriately the Gidlow Wing, and the latest addition has been mainly for the purpose of providing much-needed accommodation for the nurses. After declaring the new ward, which occupies the lower portion of the wing, open, and accepted a golden key from Mr. James Brown, chairman of the board of management, Lady Maud Wilbraham proceeded to the nurses' sitting-room, upstairs, in order to present certificates to the nurses who had recently passed their examinations. Tea was afterwards provided for the visitors. The arrangements were admirably planned by Mr. Taberner, the energetic secretary and general superintendent of the infirmary, and the proceedings went off without a hitch.

